# The Effect of β-Sheet Secondary Structure on All-β Proteins by Molecular Dynamics Simulations

**DOI:** 10.3390/molecules29132967

**Published:** 2024-06-21

**Authors:** Zhou Feng, Fang Xia, Zhouting Jiang

**Affiliations:** Department of Applied Physics, China Jiliang University, Hangzhou 310018, China; fengzhou@cjlu.edu.cn (Z.F.); xiafang1117@163.com (F.X.)

**Keywords:** molecular dynamics simulation, all-β proteins, β-sheet, secondary structure

## Abstract

The effect of β-sheet ratio and chain length on all-β proteins was investigated by MD simulations. Protein samples composed of different repeating units with various β-sheet ratios or a different number of repeating units were simulated under a broad temperature range. The simulation results show that the smaller radius of gyration was achieved by the protein with the higher proportion of β-sheet secondary structure, which had the lower nonbonded energy with more HBs within the protein. The root mean square deviation (RMSD) and the root mean square fluctuation (RMSF) both increased with temperature, especially in the case of a longer chain. The visible period was also shown according to the repeated secondary structure. Several minimum values of RMSF were located on the skeleton of Cα atoms participating in the β-sheet, indicating that it is a kind of stable secondary structure. We also concluded that proteins with a short chain or a lower ratio of β-sheet could easily transform their oriented and compact structures to other ones, such as random coils, turns, and even α-helices. These results clarified the relationship from the primary level to the 3D structure of proteins and potentially predicted protein folding.

## 1. Introduction

Proteins, as a compact molecular chain, present structural diversity because of numerous degrees of freedom in their internal organization. The biological function of a protein strongly depends on its unique structure determined by complex interactions among its residues. However, proteins with a variety of folding types can be misfolded, aggregated, or deposited in tissues, which cause diseases such as Alzheimer’s disease, type 2 diabetes mellitus, Huntington’s disease, and thyroid medullary carcinoma [1]. Understanding the structure of proteins is necessary to recognize how they work and how to enhance or suppress them [2]. Globular proteins are categorized into four structural classes, namely, all-α, all-β, α + β, and α/β proteins, based on the topology and content of their secondary structure, such as α-helices and β-sheets. All-β proteins are ubiquitous in nature and perform a wide range of functions, including the transport of hydrophobic molecules, carbohydrate recognition, enzymatic processing, and the formation of viral capsids [3]. Unlike α-helices, which are stabilized via neighborhood interactions, β-sheets are stabilized by intrastrand hydrogen bonds, involving residues that are far away along the protein sequence [4]. This property makes an isolated β strand unstable compared with an isolated α-helix. All-β proteins present many novel functions. Then, the understanding how to determine their layout is still a big challenge [3]. 

The experimental design of proteins has been considerably facilitated by the discovery of a set of rules describing constraints on the backbone geometry of the loops connecting secondary-structure elements [5]. All-β proteins, which contain appending features that are less well understood, have attracted many researchers interested in exploring their structural characteristics and folding mechanisms. Freire et al. studied the effect of strand length on the stability of parallel β-sheets. Their article reported that if the strand length of a β-sheet increased from 6 to 10 residues, the parallel β-sheet was stabilized [6]. Syud et al. investigated the effect of strand number on the stability of antiparallel β-sheets. The paper supports a cooperative, two-state model for the folding of two-stranded β-sheets and a “four-state” model for the folding of three-stranded β-sheets [7]. Chidyausiku et al. successfully designed a double-stranded β-helix based on a series of rules describing the geometry of β-arch loops and their interactions in more complex β-arcades [8]. But in these experiments, the number of protein residues and the number of strands in the all-β proteins designed by the researchers were small, and the proportion of secondary structure in the whole proteins was not discussed. The available experimental methods are still insufficient to describe the dynamic properties of proteins [9]. Therefore, simulation methods are essential to investigate the structural biology of proteins and are more and more attracting the attention of scientists [10].

With the rapid development of computer technology, computational biophysics has started to focus on researching the structure and interactions of biomolecules at the atomistic level [11,12]. Numerous fundamental questions, such as the nature of the interactions involved in protein folding, the thermodynamic states involved as a protein folds, and the structural characteristics of each thermodynamic state, still remain unanswered despite the rapid advancement of computer power and the efforts made by many researchers [13]. A recent study suggested that due to high barriers to (un)folding, a folding/unfolding equilibrium could not be established [14]. Soto et al. suggested that no significant free energy barrier separates the different conformations available [15]. Xia et al. showed that the extension of a β-sheet occurs more easily and the α-helix is more easily disrupted at high temperatures [16]. Man et al. showed that the β-strand content is important for controlling the aggregation rate [17]. Guo et al. found that non-cocoon-silk sericin contains more β-sheet structures and has better adhesive strength and hydrophilicity than cocoon-silk sericin [18]. Erickson et al. found a higher β-sheet content during its self-assembly in an increasingly hydrophilic solvent [19]. Afkhami et al. suggested that increased entanglement in samples with higher β-sheet content and longer fibril length led to higher viscosity [20]. These studies showed that the content of β-sheet is crucial in controlling the structure and dynamics of proteins and their performance of specific functions [21]. But the interplay between the synergistic and the competing effects of protein–protein and protein–water interactions and the proportion of secondary structures resulting from covalently bonded residues in a protein, which leads to special structural and dynamical responses, are still unclear [22].

In this work, we perform MD simulations of all-β proteins with different lengths or various secondary structure ratios of β-sheets. The aim of this study is to investigate the effect of β-sheets on the structural features and conformational transition of proteins. The rest of this paper is organized as follows. The simulation results, including the effects of chain length and ratio of β-sheet, are discussed in Section 2. The methodology, including the construction of a protein chain model and molecular dynamics simulation details, is presented in Section 3, and the paper is concluded in Section 4.

## 2. Results and Discussion

### 2.1. Effect of the Chain Length

In this section, the protein samples were made up of the same uncharged serine residues (SER) for modeling. The basic unit (β_6_C_6_) included a β-sheet structure composed of 6 SER residues and others, such as random coils or turns composed of 6 SER residues also. Specifically, the ratio of the β-sheet structure to others in the basic unit was fixed as 1 to 1. The protein samples including two, three, four, or five pairs of repeating units were constructed by VMD software (version 1.9.4) [23] and simulated at the temperature from 230 K to 470 K by MD method, respectively.

#### 2.1.1. Radius of Gyration

**Figure 1:**
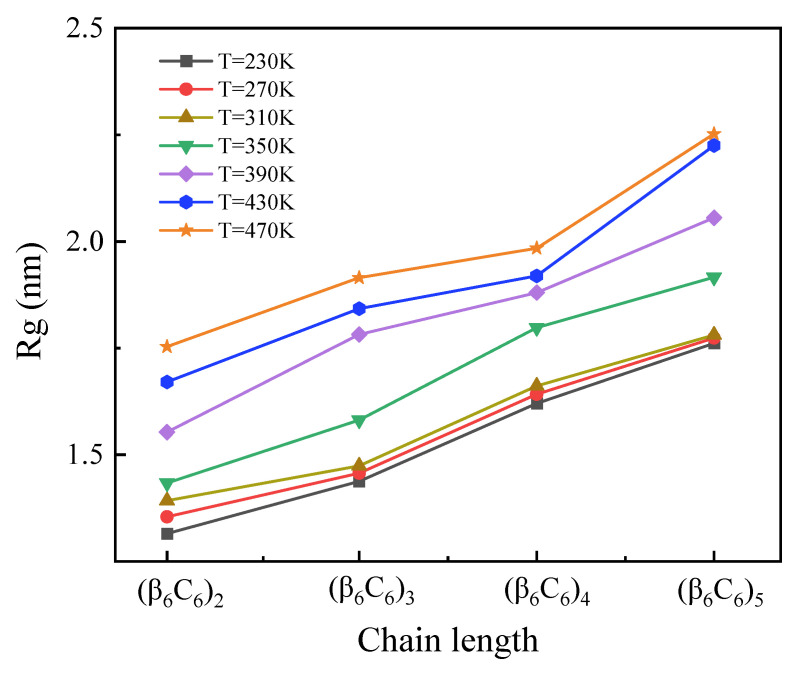
(Color online) Chain length dependence of the radius of gyration *R_g_* at variable temperatures.

The chain length dependence of the radius of gyration (*R_g_*) at variable temperatures is shown in Figure 1. It is evident that the values of *R_g_* increased linearly with the amount of residue, which constituted proteins. This change indicated the general result about the larger spatial size of longer proteins. Additionally, the *R_g_* of proteins with a certain chain length increased as the temperature increased. For the short chain, *R_g_* increased as the temperature rose uniformly. In the case of simulating longer protein samples, such a tendency was more obvious only when the temperature increased higher than 310 K. 

#### 2.1.2. Root Mean Square Deviation and Root Mean Square Fluctuation

**Figure 2:**
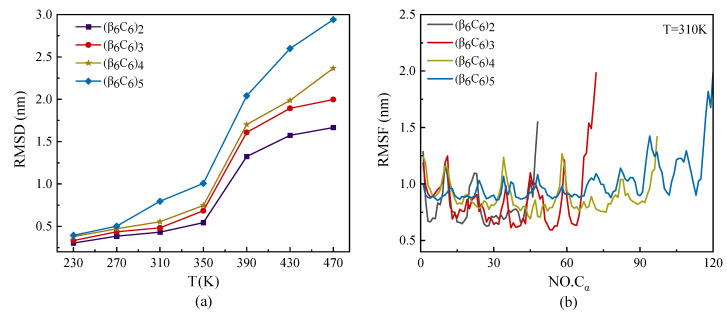
(Color online) Temperature dependence of (**a**) root mean square deviation RMSD and (**b**) root mean square fluctuation RMSF of skeleton Cα atoms at T = 310 K of protein samples with the same repeating unit but different chain lengths.

The root mean square deviation (RMSD) and root mean square fluctuation (RMSF) of protein samples with the same repeating unit β_6_C_6_, but with different chain lengths, are plotted in Figure 2. As shown in Figure 2a, the value of RMSD increased as the temperature increased. The values of the RMSD of protein samples with different lengths under a low temperature were almost the same. The samples rapidly increased as the temperature increased, especially on the condition of a large protein sample. The temperature had a higher effect on the RMSD value of a protein than its chain length. Figure 2b shows the RMSF of each protein chain under the temperature of T = 310 K. The curves of RMSF in Figure 2b show that the two ends of the protein chain had higher movement than the middle part of the protein sample. We concluded that the end parts of the protein showed various structural diversity because they had a high degree of freedom during the MD simulations. The middle part of the protein had a lower degree of freedom because of the relatively confined space occupied by the nearby atoms. Another interesting result was that the value of RMSF had the same period of vibration, which had the same repeating units. Several minimum values were located on the skeleton Cα atoms participating in the β-sheet, which indicated a kind of stable secondary structure rather than a random coil. The interval of the two adjacent minimums was about 12, which was the total amount of residue in the repeating unit.

#### 2.1.3. Secondary Structure Analysis

We estimated the effect of the chain length on the secondary structure of the protein samples according to the STRIDE algorithm implemented into the VMD software package (version 1.9.4). We analyzed the tertiary conformation of the protein by assigning different types of secondary structures to each residue based on the knowledge-based algorithm. We used the algorithm to consider the hydrogen bond energy and statistically derived the information on the torsional angles of the protein. The protein samples had identical repeating units but four different chain lengths (β_6_C_6_)_n_ (n = 2, 3, 4, and 5). The proportion of β-sheet was fixed among these protein samples. In the model of the (β_6_C_6_)_2_ system, the β-folds were located at residues 3–8, 15–20, 27–32, and 39–44. In total, two pairs of β-sheets were constructed. Similarly, the β-folds in the protein (β_6_C_6_)_4_ were located at residues 3–8, 15–20, 27–32, 39–44, 51–56, 63–68, 75–80, and 87–92, which included four pairs of β-sheets. 

**Figure 3:**
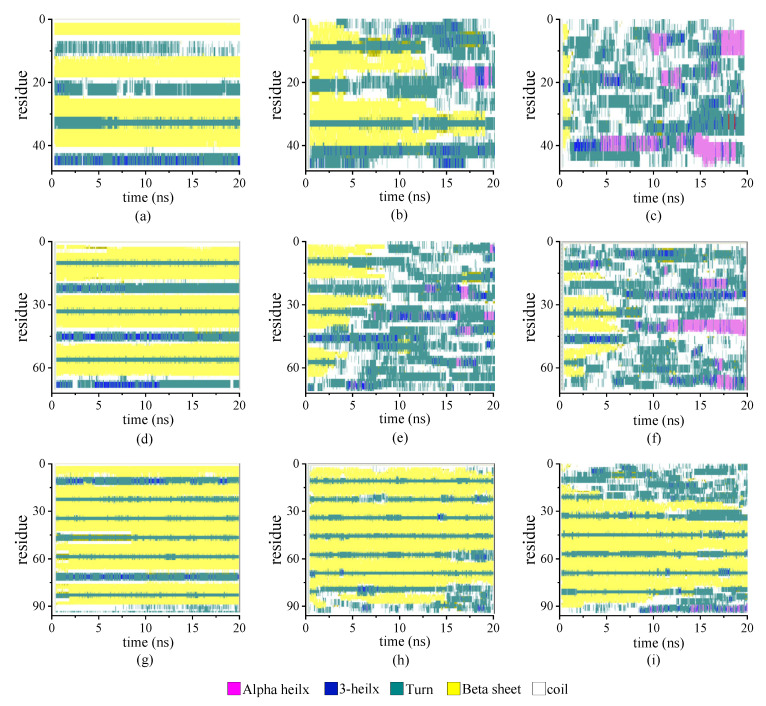
(Color online) Stride evolution of secondary structures of the protein (β_6_C_6_)_2_ at temperatures of (**a**) 230 K, (**b**) 350 K, and (**c**) 430 K; (β_6_C_6_)_3_ at temperatures of (**d**) 230 K, (**e**) 350 K, and (**f**) 430 K; and (β_6_C_6_)_4_ at temperatures of (**g**) 230 K, (**h**) 350 K, and (**i**) 430 K.

Figure 3 illustrates the stride evolution of the secondary structures in all-β protein chains (β_6_C_6_)_n_ with various chain lengths (n = 2, 3, and 4) over the simulation time. Figure 3a–3d present the secondary structure transitions of the protein chain (β_6_C_6_)_2_ under the temperatures of 230 K, 350 K, and 430 K, respectively. At a low temperature of 230 K, two pairs of β-sheets were maintained throughout the whole simulation. When the temperature increased to 350 K, the structure transitioned from the β-folds to other secondary structures, and an α-helix occurred in the first segment of the β-sheet, which exhibited minor transformation events. 

**Figure 4:**
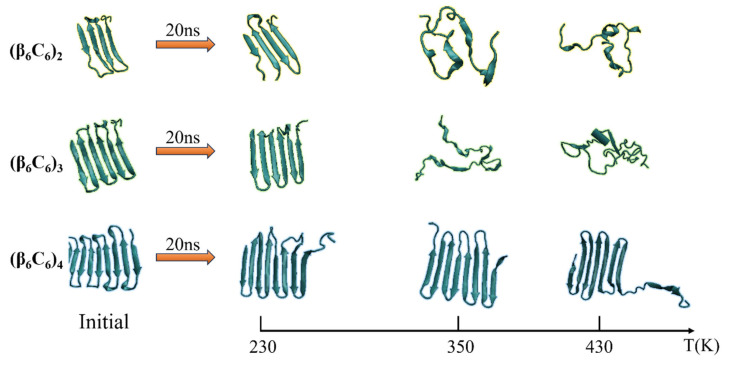
(Color online) Typical conformation of the protein (β_6_C_6_) with two, three, or four pairs at temperatures of 230 K, 350 K, and 430 K, respectively.

The second pair of β-sheets remained virtually the same. As the temperature continued to increase, additional fragments of the β-folds could not remain in the original secondary structure. The entire chain of the (β_6_C_6_)_2_ system exhibited a turn, a helix, or a random coil structure at approximate 1 ns (as shown in Figure 3c), which indicated that the rapid and drastic structural transition that appeared when the protein chain was under a high temperature. This same tendency was revealed in other protein chains. For example, the stable four pairs of β-sheet structures in (β_6_C_6_)_4_ remained unchanged during the 20 MD simulations executed at 230 K (as shown in Figure 3g). When the temperature increased to 350 K (as shown in Figure 3h), most of the β-sheet remained, except for the structural transition from the β-folds to turns or random coils at the end of the protein, where residues 87–92 of the (β_6_C_6_)_4_ occurred at 15 ns. At a temperature of 430 K (as shown in Figure 3i), in addition to the end, the beginning part of the system (β_6_C_6_)_4_, residues 1–20 started to change its secondary structure within 2 ns. Compared with the results of the protein samples shown in Figure 3, the same tendency was evident that the structural transition occurred on the protein samples once the temperature was high enough. Another interesting finding was that the transition temperature was different according to the chain length. Focusing on the results shown in Figure 3c,f,i, when three protein samples (β_6_C_6_)_n_ (n = 2, 3, and 4) were simulated at a temperature of 430 K, the structural transition occurred within 1 ns in the (β_6_C_6_)_2_ system. A similar event occurred after simulating for 5 ns in the (β_6_C_6_)_3_ protein. In the case of the (β_6_C_6_)_4_ protein at 430 K, most of β-sheets remained, except for the ends of the protein chain, which had greater movement than the middle part of the protein sample. The transition temperature of the secondary structure of the entire (β_6_C_6_)_4_ protein should be higher than 430 K. We concluded that it was difficult for the longer protein chain to transform its secondary structure based on the effect of the temperature. The beginning or the end of the protein sample changed its secondary structure earlier than the middle part during the MD simulation, which indicated greater structural instability than the intermediate part of the protein chain. The typical conformations of these all-β proteins (β_6_C_6_) with two, three, or four pairs at temperatures of 230 K, 350 K, and 430 K are shown in Figure 4. The results clarified the relationship between chain length and temperature.

### 2.2. Effect of the β-Sheet Proportion

The protein samples were made up of uncharged SER residue of the same length (120 residues) but with different β-sheet proportions in the repeating unit. The ratio of the β-sheet to other secondary structures (random coils or turns) in the basic unit was set as 0.25:1, 0.5:1, 0.67:1, 1:1, or 2:1 in its initial configuration. We selected the protein samples (β_3_C_12_)_4_, (β_5_C_10_)_4_, (β_8_C_12_)_3_, (β_6_C_6_)_5_, and (β_8_C_4_)_5_ to investigate the effect of the β-sheet proportion on the structural characters of proteins. We selected the protein chains with a fixed amount of residue as the simulated samples for the MD simulation at a temperature range from 230 K to 470 K.

#### 2.2.1. Energy

**Figure 5:**
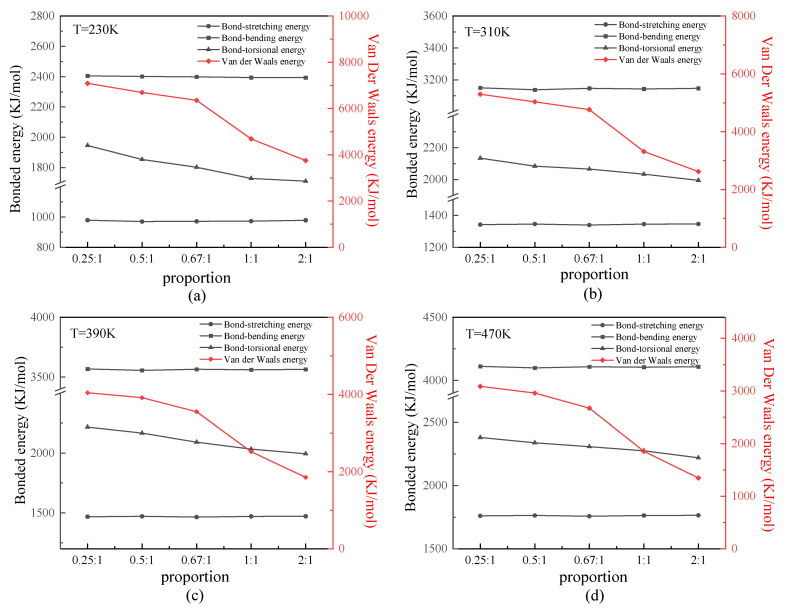
(Color online) Relationship between the bonded or van der Waals energy and β-sheet proportion in the repeating unit of protein chains at temperatures of (**a**) 230 K, (**b**) 310 K, (**c**) 390 K, and (**d**) 470 K.

We analyzed the bonded and nonbonded energies of the protein samples. Because all protein samples were constructed by uncharged SER, van der Waals energy is discussed primarily as nonbonded energy. Figure 5 shows the relationship between the bonded or van der Waals energy and β-sheet proportion in the repeating unit of the protein chains under different temperatures. As shown in these four figures, the tendency was that the bond-stretching and bond-bending energy of any of the protein samples presented close values at certain temperatures. This result demonstrated that the bond-stretching and bond-bending energy were basically unaffected by the secondary structure of the protein chain, which depended on its first structure (i.e., chain length only). In contrast, the bond-torsional energy slightly decreased with the increasing proportion of the β-sheet in the repeating unit. Meanwhile, the van der Waals energy was most affected by the secondary structure, as it significantly decreased with an increase in the proportion of β-sheet. From an energetic standpoint, the lower energy indicated a more stable structure, such as the β-folds in this work, because of the existence of more two-pair atom interactions along the β-folded structure. This result indicated that the nonbonded interaction plays an important role in the stability of the protein structure. As shown in these four figures, the difference was that the absolute values of all types of energy varied according to the temperature. In detail, the bonded energy, including bond-stretching, bond-bending, and bond-torsional energies, increased linearly as the temperature increased. This was proven by the theorem of energy equipartition—that is, the average energy associated with each individual degree of freedom (bond length/bond angle/dihedral angle) was linearly proportional to the system temperature. The van der Waals energy, however, decreased as the temperature increased, which was the same result found in our previous work [24,25,26].

#### 2.2.2. Structural Characteristics

**Figure 6:**
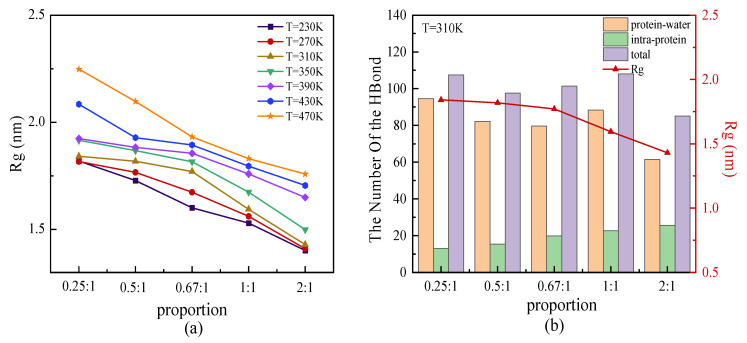
(Color online) β-sheet proportion dependence of (**a**) radius of gyration of protein chains under different temperatures, and (**b**) number of HBs with respect to the radius of gyration of the protein at T = 310 K.

We simulated the protein samples with β-sheet proportions of 0.25:1, 0.5:1, 0.67:1, 1:1, and 2:1 at temperatures ranging from 230 K to 470 K. The radius of gyration *R_g_* as the function of β-sheet proportion is shown in Figure 6a. The results showed that the radius of gyration for each protein mainly increased with the temperature. At a certain temperature, the protein with a higher proportion of β-sheet had a small size, which indicated the more compact 3D structure of the protein with the same chain length. Hydrogen bonds (HBs) played a significant role in stabilizing the secondary structure of the proteins. The correlation of total hydrogen bonds, including intra-protein and water-protein types, with the proportion of β-sheet is plotted in Figure 6b. As shown in Figure 6b, the number of water-protein HBs was several times higher than that of intra-protein HBs. The change in the total number of HBs was dominated by the numerical variation in the water-protein HBs. Although it had a low correlation between the total number of HBs and the proportion of β-sheet, the proportion dependency of intra-protein HBs showed the clear tendency that it increased in number along with the increasing proportion of β-sheet in the repeating unit. This result indicated that more intra-protein HBs formed along the parallel β-folded structures. Contrary to the changes in the intra-protein HBs as a function of the β-sheet proportion, the smaller *R_g_* of the protein with a higher proportion of β-sheet is also shown in Figure 6b. The opposite trend between the number of intra-protein HBs and the value of *R_g_* as a function of the β-sheet proportion indicated that the more β-sheet segments presented a relatively small size of the protein, with more hydrogen bonds acting on the interior of the protein. This result confirmed that HBs, especially the intra-protein types, played a crucial role in promoting protein stability.

**Figure 7:**
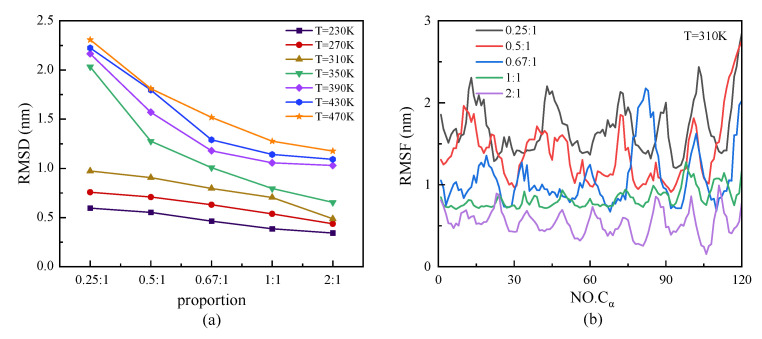
(Color online) β-sheet proportion dependence of (**a**) RMSD and (**b**) RMSF of protein chains with different β-sheet proportions at T = 310 K.

We investigated the structural stability by analyzing the parameters of RMSD and RMSF, which are shown in Figure 7. At a certain temperature, the protein with a higher proportion of β-sheet presented a steady characteristic conformation with a low value of RMSD—that is, the value of RMSD decreased with the increasing proportion of β-sheet. Meanwhile, the curves of RMSD presented an increasing tendency with temperature, especially in the case of the protein with a lower proportion of β-sheet at a high temperature range. The relationship between the root mean square fluctuation (RMSF) of the skeleton Cα atoms and the change in β-sheet proportion in the repeating unit of protein chains under T = 310 K is shown in Figure 7b. The results showed the same tendency that the protein with a lower proportion of β-sheet had a higher value of RMSF, which revealed the higher degrees of freedom of the skeleton Cα atoms in the protein. The inverse correlation of the β-sheet ratio and the RMSF indicated that the structural stability of the protein was weakened with a decrease in the β-sheet ratio. In Figure 7b, the curves of RMSF show the specific period related to the ratio of β-sheet in the repeating unit. The trough of the curve corresponded to the β-sheet region, which indicated that the secondary structure was more stable than the random coil or turns. In the model of the protein sample with a β-sheet proportion of 0.25:1, however, the RMSF value of the residue Cα atom that originally belonged to the β-sheet structure did not decrease as significantly as the other protein samples, which indicated the transfer of the β-folded structure to other types at the temperature T = 310 K.

Figure 6 and Figure 7 show that the overall size and instability of the protein were positively correlated with temperature. Then, a protein with a low percentage of β-sheets exhibited a visible change of its structural size by temperature. It implies that proteins with a low ratio of β-sheets are more flexible to alter their structure in higher temperature quickly. But more β-sheets in the protein can provide a more temperature-resistant and generally stable structure. In short, the protein with more secondary structures of β-sheet presents a more compact structure with higher stability.

#### 2.2.3. Secondary Structure Analysis

**Figure 8:**
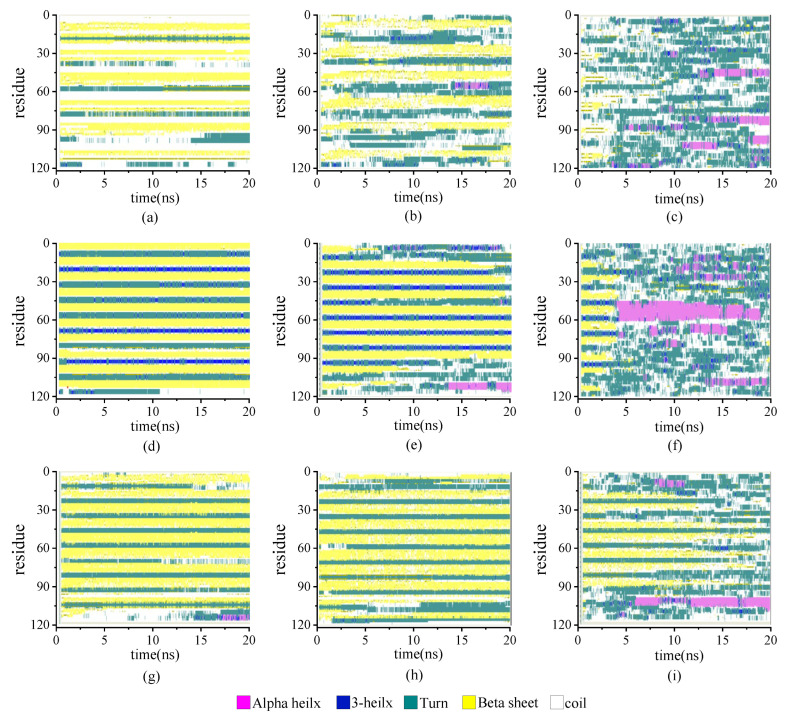
(Color online) Stride evolution of secondary structures of the protein samples with the β-sheet proportion of 0.67:1 at temperature of (**a**) 230 K, (**b**) 350 K, and (**c**) 430 K; 1:1 at temperature of (**d**) 230 K, (**e**) 350 K, and (**f**) 430 K; and 2:1 at temperature of (**g**) 230 K, (**h**) 350 K, and (**i**) 430 K.

**Figure 9:**
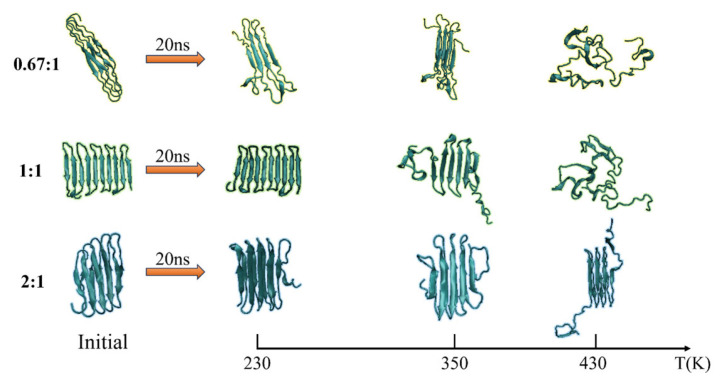
(Color online) Typical conformation of the protein samples with the β-sheet proportion of 0.67:1, 1:1, and 2:1 at temperature of 230 K, 350 K and 430 K, respectively.

The secondary structure evolutions of protein samples are shown in Figure 8. We obtained the time evolution of the secondary structure from the protein samples with the same chain length but with different β-sheet proportions in the repeating unit as 0.67:1, 1:1, or 2:1 under the temperature T = 230, 350, and 430 K, respectively. In keeping with the results shown in Figure 3, the temperature dependency of the structural transition is clearly presented from the left column (T = 230 K) to the right column (T = 430 K). For example, the secondary structure evolutions of the protein sample (β_6_C_6_)_5_ are shown in Figure 8d–f from 230 K, 350 K, to 430 K. The half-ratios of the β-sheets over the whole protein chain were located on the 3–8, 15–20, 27–32, 39–44, 51–56, 63–68, 75–60, 87–92, 99–104, and 111–116 residues. When the protein was at a low temperature of 230 K, the β-sheets remained stable during the entire simulation process. When the protein (β_6_C_6_)_5_ was at T = 350 K, both ends of the protein chain started to change its secondary structure from β-folds to other structures, and even became more stable α-helix structures at 13 ns. When the temperature kept increasing, the drastic secondary structure transition happened. All five pairs of β-sheets in the protein (β_6_C_6_)_5_ transferred to turn, random coil, and helix within 5 ns. This result clearly showed that the higher temperature led to the quick and drastic structural transitions. In contrast, at a certain temperature, the protein with higher β-sheet proportions could retain the β-folds for a longer time. In Figure 8c, the protein sample (β_8_C_12_)_3_ with the β proportion of 0.67:1 showed that the β-sheet structure could not be retained. The structural transition from β-sheets to other types, such as random coils, turns, and even α-helices within 1 ns, indicated a violent change in the protein (β_8_C_12_)_3_ at a temperature of 430 K. The structural transition of the protein chain (β_8_C_4_)_5_ with the highest proportion of 2:1 at a temperature of 430 K is shown in Figure 8i. Unlike the tendency shown in Figure 8c, even at such a high temperature, the protein chain (β_8_C_4_)_5_ still kept the original secondary structure, especially in the middle part of the protein chain, except when two ends of the chain (residues 2–9, 98–105, and 110–117) began to transfer randomly from β-folds into helices, coils, and corner structures. Most of the structures were well maintained for the 18 ns simulation. We concluded that the protein chain constructed by a higher β-sheet proportion presented a stable secondary structure with less of a temperature effect. The typical conformations of these all-β proteins with a β-sheet proportion of 0.67:1, 1:1, and 2:1 at the temperature of 230 K, 350 K, and 430 K are shown in Figure 9. This showed that the relationship between the ratio of secondary structures and the temperature was intuitive.

## 3. Materials and Methods

Each protein sample was made up of the same uncharged residue serine (SER) for modeling. Its sequence, as the primary structure of the protein sample, was connected by residue SER. The basic unit included a β-sheet structure and others, such as the random coil and turns, in the present study were constructed by VMD software (version 1.9.4). In the basic unit, the proportion of the β-sheet structure varies, but the amount of amino acids in the unit is the same. The protein samples selected in this work were mainly the following two cases: (1) the protein chains including 2, 3, 4, or 5 pairs of repeating units (β_6_C_6_), containing 6 residues to construct β-folds and 6 residues as others. The protein chains were made up of 46, 72, 96, and 120 SER residues, respectively. (2) The protein chains were made up of 120 residues with the ratio of the β-sheet to other secondary structure in the repeating unit as 0.25:1, 0.5:1, 0.67:1, 1:1, 2:1, respectively. The proteins (β_3_C_12_)_4_, (β_5_C_10_)_4_, (β_8_C_12_)_3_, (β_6_C_6_)_5_, and (β_8_C_4_)_5_ were selected to investigate the effect of the β-sheet proportion on the structural characters of proteins. The protein samples were simulated at temperatures from 230 K to 470 K by MD method.

All simulations were performed using molecular dynamic algorithms implemented in an NAMD 2.6 software package (Beckman Institute, University of Illinois at Urbana-Champaign, Urbana, IL, USA) with an all-atom CHARMM27 force field [27]. The energy-minimized structures were firstly solvated using TIP3P water in a cubic box with 2 nm thickness around the protein. The periodic boundary conditions were applied. The simulated system was energy minimized for 60,000 steps followed by 120 ps of pressure equilibration. Then, the molecular dynamics simulations were carried out for 20 ns under the condition of a certain temperature and constant pressure P = 1 atm. The temperature was set in a step of 40 K from 230 K to 470 K. The time step and mesh spacing were set as 2 fs and 0.1 nm, respectively. The VDW interaction involved switching functions with a cutoff distance of 1.2 nm (staring at 1 nm). The simulated system interacted by the nonbonded potentials and bonded potentials, including bond-stretching, bond-bending, and bond-torsional potentials.

The radius of gyration, *R_g_*, is a basic measurement of the overall size of a chain molecule, which is defined as

(1) $$R_{g}=\sqrt{\frac{1}{N}\sum_{i=1}^{N}{\left|r\left(i\right)-r_{center}\right|}^{2}}$$

where *N* is the number of protein atoms. *r*(*i*) and *r_center_* are the coordinates of an atom *i* and the center of mass, respectively. The conformational stability of proteins during the simulation procedure was examined by calculating the root mean square deviation (RMSD) and root mean square fluctuation (RMSF). The RMSD is a numerical measurement of the conformational changes between two structures. It is defined as

(2) $$\mathrm{RMSD}=\sqrt{\frac{1}{N}\sum_{i=1}^{N}{\left|r_{final}\left(i\right)-r_{initial}\left(i\right)\right|}^{2}}$$

where *N* is the number of protein atoms. *r_final_*(*i*) and *r_initial_*(*i*) are the coordinates of an atom *i* in its final structure and initial structure, respectively. The RMSF represents the fluctuation of the coordinates of each atom of the protein near its reference coordinate during the simulation process. It is an important tool to characterize the freedom of the center *Cα* atoms in protein chains, which is defined as

(3) $${\mathrm{RMSF}}_{i}=\sqrt{\frac{1}{t_{total}}\sum_{t_{j}=1}^{t_{total}}{\left|r_{i}\left(t_{j}\right)-r_{i}^{ref}\right|}^{2}}$$

where *t_total_* is the total simulation time, and the reference coordinate, *r^ref^*, is the average coordinate of the *Cα* atom during the whole simulation period.

## 4. Conclusions

In this article, MD simulations were performed on the protein samples with the different chain lengths or β-sheet proportions at a wide range of temperatures. We investigated the potential energy and structural parameters such as radius of gyration, the root mean square deviation, and the root mean square fluctuation, as well as the evolution of secondary structures to present the effect of the β-sheet secondary structure on the all-β proteins. The current results indicate the following:

(1) The radius of gyration, *R_g_*, increased with the chain length as well as the temperature. But it had the negative effect of the β-sheet proportions, i.e., in the protein with the same chain length but higher proportion of the β-sheet secondary structure, the lower *R_g_* observed indicated the compact structure.
(2) The root mean square deviation, RMSD, of different chain lengths at various temperature showed that RMSD increased with temperature, especially in the case of the longer chain. Meanwhile, the longer chain presented the higher value of RMSF at a certain temperature. The visible period was also shown according to the repeated secondary structures. Several minimum values of RMSF were located on the skeleton Cα atoms participating in the β-sheets, which indicated a kind of stable secondary structure rather than random coil.
(3) Comparing the protein chains with the same length at the certain temperature, the protein with the higher proportion of the β-sheet structure in a repeating unit presented the lower nonbonded energy with more HBs within the protein, the smaller *R_g_,* as well as the small value of RMSD. It also concluded that the protein with the lower ratio of β-sheet could easily transform its oriented and compact structures to others, such as random coils, turns, and even α-helices.

## Data Availability

Data are contained within the article.
